# The Association between Total Protein and Vegetable Protein Intake and Low Muscle Mass among the Community-Dwelling Elderly Population in Northern Taiwan

**DOI:** 10.3390/nu8060373

**Published:** 2016-06-17

**Authors:** Ru-Yi Huang, Kuen-Cheh Yang, Hao-Hsiang Chang, Long-Teng Lee, Chia-Wen Lu, Kuo-Chin Huang

**Affiliations:** 1 Department of Medical Education, E-Da Hospital and I-Shou University, Kaohsiung City 82445, Taiwan; Ruyi@mail.harvard.edu; 2 Department of Family Medicine, National Taiwan University Hospital, Taipei 10002, Taiwan; kcyang@ntuh.gov.tw (K.-C.Y.); allanchanghs@gmail.com (H.-H.C.); ltlee@ntuh.gov.tw (L.-T.L.); biopsycosocial@gmail.com (C.-W.L.); 3 Department of Community and Family Medicine, National Taiwan University Hospital, Hsin-Chu Branch, Hsinchu City 30059, Taiwan; 4 Department of Family Medicine, National Taiwan University Hospital, Bei-Hu Branch, Taipei 10845, Taiwan; 5 Graduate Institute of Clinical Medical Science, China Medical University, Taichung 40402, Taiwan

**Keywords:** sarcopenia, protein, vegetable protein, nutrition, vegetarian diet

## Abstract

Sarcopenia, highly linked with fall, frailty, and disease burden, is an emerging problem in aging society. Higher protein intake has been suggested to maintain nitrogen balance. Our objective was to investigate whether pre-sarcopenia status was associated with lower protein intake. A total of 327 community-dwelling elderly people were recruited for a cross-sectional study. We adopted the multivariate nutrient density model to identify associations between low muscle mass and dietary protein intake. The general linear regression models were applied to estimate skeletal muscle mass index across the quartiles of total protein and vegetable protein density. Participants with diets in the lowest quartile of total protein density (<13.2%) were at a higher risk for low muscle mass (odds ratio (OR) 3.03, 95% confidence interval (CI) 1.37–6.72) than those with diets in the highest quartile (≥17.2%). Similarly, participants with diets in the lowest quartile of vegetable protein density (<5.8%) were at a higher risk for low muscle mass (OR 2.34, 95% CI 1.14–4.83) than those with diets in the highest quartile (≥9.4%). Furthermore, the estimated skeletal muscle mass index increased significantly across the quartiles of total protein density (*p* = 0.023) and vegetable protein density (*p* = 0.025). Increasing daily intakes of total protein and vegetable protein densities appears to confer protection against pre-sarcopenia status.

## 1. Introduction

Sarcopenia, the loss of muscle mass that occurs with aging, was first described by Rosenberg in 1989 [1]. Muscle mass decreases at a rate of 3% to 8% per decade after the age of thirty, and this loss accelerates further in older age [2]. Sarcopenia drastically increases the risk of fractures, which leads to functional impairment in daily activities and incurs a huge economic burden in the form of prolonged hospitalization and rehabilitation [3,4,5,6,7]. Several factors have been reported to potentially contribute to sarcopenia, namely genetics, dietary protein intake, physical inactivity [8], neurodegenerative disease, hormone deficiency, and subclinical inflammation [9].

Because muscle mass decreases with aging, higher protein intake is suggested to maintain nitrogen balance in the elderly [10,11,12]. As sarcopenia is precipitated by a negative muscle protein balance [13], adequate protein intake is theoretically crucial to the maintenance of muscle mass. However, the effects of dietary protein supplement intervention on lean muscle mass remains unclear [13,14,15,16,17]. Among long-term observational studies, the relationships between dietary protein intake and the loss of muscle mass were inconsistent [18,19,20,21,22,23]. Furthermore, the association between vegetable protein intake and muscle mass in the elderly remains unclear. While muscle mass improved significantly with increased animal protein intake in one observational study [20], other comparisons between animal and vegetable protein combined with resistance training have yielded conflicting results [24,25]. No study has examined whether vegetable protein is associated with muscle mass in an Asian population.

The aims of this study were to determine if an association existed between dietary protein intake and low muscle mass (LMM), and if vegetable protein intake was associated with LMM.

## 2. Materials and Methods

### 2.1. Study Population

A total of 327 ambulatory volunteers living in the community of Taipei, Taiwan, were enrolled through flyers and word-of-mouth in 2007. Subjects were eligible if they could complete the body composition measurement and questionnaire with a trained interviewer and were excluded if they had end-stage organ diseases. The completion rate of the questionnaire survey was 100%. All participants provided written informed consent. All protocols were approved by the Ethics Committee of the National Taiwan University Hospital (IRB NO: 200701035R).

### 2.2. Body Composition

Skeletal mass was estimated by bioelectrical impedance analysis (BIA) (Tanita BC-418, Japan) [26]. Bioelectrical resistance was measured using 8 TANITA BC-418 electrodes with a frequency of 50 kHz at 500 uA. Electric current was supplied from the electrodes on the tips of the toes of both feet and the fingertips of both hands. Subjects stepped on the weighing platform in bare feet with heels on the posterior electrodes and the front part of the feet in contact with the anterior electrodes. They stood still without bending knees. The subjects also grasped the grips with both hands. When the measurements were done, the analyzer displayed resistance. Skeletal mass was estimated from Skeletal Mass (kg) = (0.401 × (height)^2^/resistance + (3.825 × gender) − (0.071 × age) + 5.102) [26]. Skeletal muscle mass index (SMI) was obtained by dividing skeletal mass by height squared. Low muscle mass (LMM) was defined as an SMI of two standard deviations or more below the gender-specific mean for the 18–40 years old Taiwanese population (SMI < 8.87 kg/m^2^ in men and SMI < 6.42 kg/m^2^ in women) [26,27].

### 2.3. Dietary Assessment

Participants completed a food frequency questionnaire (FFQ) with a registered dietitian. The FFQ food list was derived from the Elderly Nutrition and Health Survey in Taiwan [28] and validated with a 24-h recall method [29]. The FFQ was analyzed for macronutrients and micronutrients using estimated data from the Taiwan Food and Drug Administration [30]. Nutrient density, the percentage of total energy from proteins, lipids, and carbohydrates, was calculated separately. Total proteins included animal and vegetable proteins. Vegetable proteins were categorized into major food groups including beans, vegetables, and staples. The questionnaire also included current use of vitamin-mineral supplements, *i.e.*, multivitamins, vitamin B complex, and calcium supplements at least once weekly.

### 2.4. Demographics, Lifestyle Factors, and Nutritional Biomarkers

Information about age, gender, anthropometric data, cigarette use, alcohol use, exercise, chronic disease history [31], and current use of nutritional supplements [32] was obtained via the questionnaire at baseline. Current smokers were defined as those who had smoked for more than six months. Former smokers were defined as those who had quit smoking at least one year before. Former smokers and never smokers were grouped together as non-current smokers for further analysis. Current drinkers of alcohol were defined as those who had drunk at least one ounce of alcohol per week for six months. Former drinkers were defined as those who had quit drinking at least one year before. Former drinkers and never drinkers were grouped together as non-current drinkers for further analysis. Anthropometric measurements, including height and weight, were performed using a standard stadiometer. Waist was measured through the umbilicus and midpoint between the subcostal border and the iliac crest. Body mass index was calculated as weight (kg) divided by height squared (m^2^). An eight-hour fasting venous blood sample was drawn from each participant. A complete blood count with differential and biochemistry panels including serum glucose, total cholesterol, high-density lipoprotein (HDL) cholesterol, LDL cholesterol, and triglycerides were assessed with an automatic spectrophotometric assay (HITACHI 7170, Japan). Fasting glucose was determined by a glucose oxidase method, and serum triglycerides were determined with a free glycerol banking method. HDL and LDL were determined by chemical modified enzyme and sodium *N*-(2-Hydroxy-3-sulfopropyl)-3,5-dimethoxyaniline (HSDA).

### 2.5. Statistical Analysis

After testing the normal distribution with the Kolmogorov–Smirnov test, either the independent t-test or Wilcoxon rank sum test was used based on an underlying distribution for continuous variables, and the chi-square test was used for categorical variables in baseline characteristics and nutritional parameter analysis. The associations between LMM and total protein or vegetable protein density were analyzed by using the “multivariate nutrient density models” [33], in which nutrient density and total energy were fitted as independent variables in multivariate logistic regression models. This was an isocaloric model emphasizing the effect of dietary composition after making adjustments for differing body size, physical activity, and metabolic requirements. Total protein density and vegetable protein density were evaluated as categorical variables by quartiles: the quartiles of total protein density were separated at 13.2%, 15.0%, and 17.2%, and the quartiles of vegetable protein density were separated at 5.8%, 7.5%, and 9.4%. Potential confounders including age, gender, BMI, waist, albumin, hemoglobin, lymphocyte percentage, total energy, smoking, alcohol use, physical activity, diabetes mellitus, and hypertension were chosen based on literature reviews [34,35,36] and subjective knowledge. We added total protein as one of the confounders of vegetable protein in the regression model and also tested the interaction between total protein and vegetable protein. Nutrient densities in the LMM and normal groups were estimated by the least square (LS) means after adjustment for potential confounders. The LS means of the SMI in each quartile of the total protein density group and the vegetable protein density group were compared. Statistical analyses were performed using PASW statistical software (18th version, IBM SPSS statistics).

## 3. Results

The characteristics of the study population are shown in Table 1. The participants’ ages ranged from 65 to 85 without a significant difference between the LMM and normal groups (*p* = 0.688). The LMM group exercised less than did the normal group (*p* = 0.019) and also had more participants with diabetes mellitus (*p* = 0.036). Other nutritional biomarkers, major diseases, and lifestyle habits revealed no differences.

The dietary intake of total calories and macronutrients is shown in Table 2. Total protein density and vegetable protein density were significantly lower in the LMM group than in the normal group (total protein density 14.5% ± 2.9% *versus* 15.5% ± 3.1%, *p* = 0.007 and vegetable protein density 7.0% ± 2.4% *versus* 8.1% ± 3.0%, *p* = 0.004). The fat intake and fat density were slightly higher in the LMM group compared to the normal group (*p* = 0.012, *p* = 0.028 respectively). No significant differences were found in animal protein, total energy, carbohydrates, and vitamin-mineral supplements.

Odds ratios (OR) for LMM among the different total protein and vegetable protein quartiles are shown adjusted for confounders in different models in Table 3. Participants in the lowest total protein density quartile (<13.2%) had a higher risk for LMM than those in the highest quartile (>17.2%) (OR: 3.03, 95%CI: 1.37–6.72). Participants in the lowest vegetable protein density quartile (<5.8%) had a higher risk for LMM than those in the highest quartile (>9.4%) (OR: 2.34, 95%CI: 1.14–4.83) in the multivariate nutrient density model. The result of interaction between total protein and vegetable protein density was not statistically significant.

**Figure 1:**
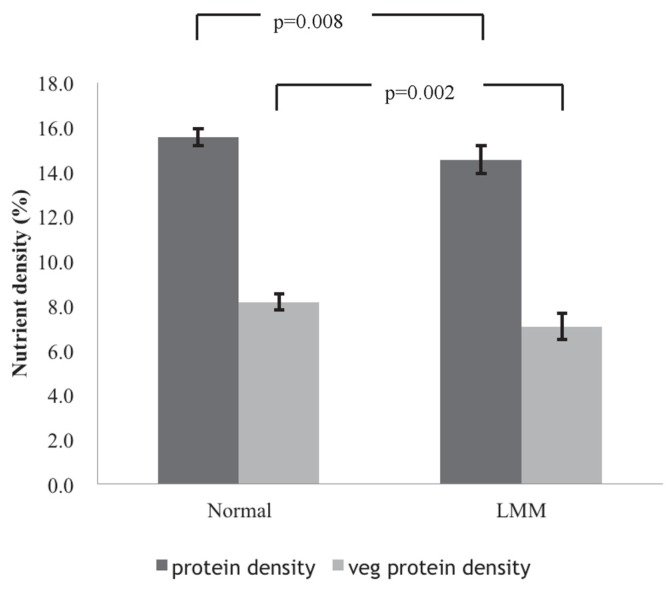
The adjusted least square (LS) means with 95% CI of total protein density and vegetable protein density the between low muscle mass (LMM) and normal groups.

**Figure 2:**
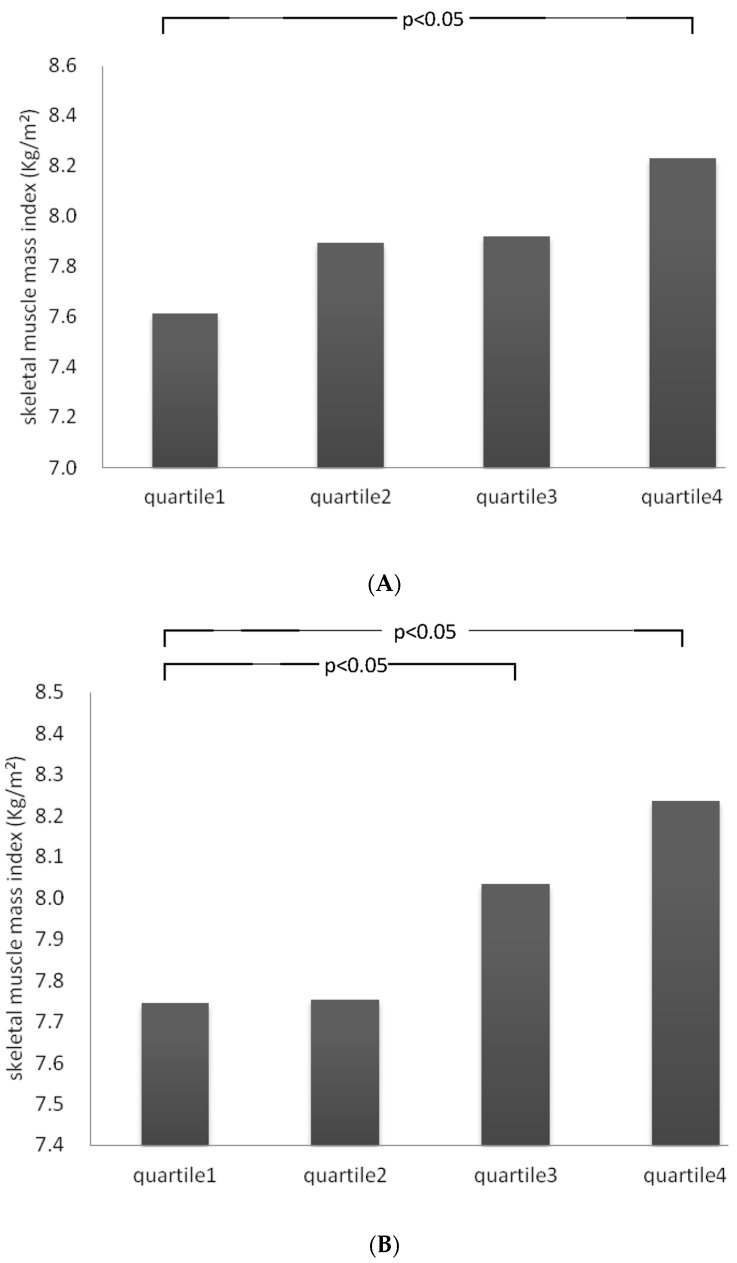
The adjusted least square (LS) means with 95% CI for skeletal muscle mass index (SMI) among the different quartiles of total protein density (**A**) and vegetable protein density (**B**).

After adjustments for the confounding factors of total energy, fat density, age, gender, BMI, waist, albumin, hemoglobin, lymphocyte count, smoking, alcohol use, exercise, diabetes mellitus, and hypertension, the LS means of the total protein density (LMM:14.5%, normal:15.5%) and vegetable protein density (LMM:7.0%, normal:8.2%) were significantly lower in the LMM group compared to the normal group (*p* = 0.008 and *p* = 0.002, respectively) (Figure 1). The LS means of SMI showed significantly increasing trends among the four groups for both total protein density (*p* for trend = 0.023, Figure 2A) and vegetable protein density (*p* for trend = 0.025, Figure 2B) after adjustment for the same confounding factors.

The LS means of total protein density (14.5% *vs.* 15.5%, *p* = 0.008) and vegetable protein density (7.0% *vs.* 8.2%, *p* = 0.002) were compared between the LMM and normal groups after adjusting for total energy, fat density, age, gender, BMI, waist, albumin, hemoglobin, lymphocyte, smoking, alcohol use, exercise, diabetes mellitus, and hypertension.

The LS means of SMI in the quartiles of total protein density (<13.2%, 13.2%~15.0%, 15.0%~17.2% and ≥17.2%) and vegetable protein density (<5.8%, 5.8%~7.5%, 7.5%~9.4% and ≥9.4%) were calculated after adjusting for total energy, fat density, age, gender, BMI, waist, albumin, hemoglobin, lymphocyte, smoking, alcohol use, exercise, diabetes mellitus, and hypertension. The LS means of SMI increased with the quartile increments of total protein density (*p* for trend: 0.023) and the LS means of SMI also increased with the quartile increments of vegetable protein density (*p* for trend: 0.025).

## 4. Discussion

We here demonstrated that pre-sarcopenia status was associated with a low intake of total protein density and vegetable protein density in the community-dwelling elderly population. Participants with the lowest mean total protein intake (11.7%) had an almost threefold risk for LMM compared to those with the highest mean protein intake (18.3%). Participants with the lowest mean vegetable protein intake (4.9%) also had an over twofold risk for LMM compared with those with the highest mean vegetable protein intake (10.9%). Furthermore, the SMI increased along with increments in total protein or vegetable protein intake. Higher protein and vegetable protein intake remained associated with higher muscle mass after adjusting for confounders.

The total protein intake in our study was compatible with the median protein intake of 15% in the United States. This exceeded the Recommended Dietary Allowance (RDA) of 0.8 g/kg/day of protein (*i.e.*, 56 g of protein/day for a 70-kg person, or 11% of total energy assuming a 2000-kcal/day diet). However, the RDA-estimated requirements were based on the avoidance of negative nitrogen balance, not on the prevention of functional decline in the aging population [37]. In our study, the healthy community-dwelling elderly participants with the lowest mean intake of total protein density (11.7%) had a threefold risk for LMM compared with those with the highest mean intake of total protein density (18.3%). This result supported the European Union Geriatric Medicine Society (EUGMS)’s recommendations for increasing dietary protein intake from 0.8 g/kg/day (11%) to a range of at least 1.0–1.2 g/kg/day (14%–16.8%) [38]. Furthermore, the Society for Sarcopenia, Cachexia, and Wasting Disease also recommended that the level of protein intake be 1 to 1.5 g/kg/day (14%–21%) to prevent and mitigate sarcopenia [39].

Participants with greater total protein intake had greater skeletal muscle mass. Few interventional studies have shown that adequate protein intake could prevent lean muscle loss; two of the studies were conducted among the subjects who were malnourished [14,15], and other studies were limited by a small sample size or a short-term intervention period [13,16]. Longitudinal studies showed that higher protein intake was associated with less lean muscle loss [18,19,20,21]. However, those studies included middle-aged adults [21] or used imprecise measures of lean muscle mass such as the measurement of the mid-arm muscle area [18]. Cross-sectional studies showed conflicting results for the association between nutrition and lean muscle mass in the elderly [22,23]. Our study analyzed the relationship between LMM and protein intake with an appropriate adjustment for lifestyle and metabolic markers.

We also found a significant association between vegetable protein and LMM, albeit a much smaller association with animal protein intake. A three-year follow-up study of an elderly cohort showed a significant association between animal protein intake and accretion on lean muscle mass [20]; however, a greater proportion of animal protein intake than vegetable protein at baseline might have limited the statistical significance. Furthermore, compared with a meat containing diet, a lacto-ovo vegetarian diet combined with the same extent of resistance training promoted lean mass in older men in one study [24], but not in another [25]. The mixed results could be attributed to the use of the hydrostatic weighting method for body composition and a higher protein intake in the mixed-diet arm than in the lacto-ovo vegetarian diet arm. In contrast, our LMM and normal groups had a similar proportion of animal protein density (7.5 ± 2.6 *vs.* 7.4 ± 2.7, *p* = 0.789). This made it possible to test the differences in vegetable protein between the two groups.

Several possible mechanisms for the effect of vegetable protein on muscle mass have been proposed. First, obtaining adequate essential amino acids is necessary to prevent sarcopenia. The quality of vegetable protein depends on the food source and could have an equivalent nutrition value as animal protein [40]. Second, legumes such as soybeans and cowpeas have high levels of leucine, which increases protein anabolism and decreases protein breakdown [41,42]. Finally, leucine and its metabolite, β-hydroxy-β-methylbutyrate, have been considered as important components in combating sarcopenia [41,42]. Since the elderly have distinct modes of muscle protein synthesis in response to different sources of protein compared with the younger group [43,44], our study focus mainly with elderly participants was better at estimating the preventive strategy of sarcopenia. The widely available vegetable protein in Taiwanese foods comes from rice, various vegetables (e.g., Chinese spinach, Chinese kale, Chinese leeks, bamboo shoots, loofah), beans (e.g., red, black, and kidney beans), and snacks (e.g., tofu and soy pudding) that differ from conventional Western diets; this might explain our unique findings among the Chinese Asian population.

Our study has limitations. First, this study adopted the FFQ, which may predispose the participants to measurement errors. Although the estimates of association between LMM and dietary protein could be weakened, a multivariate nutrient density model could avoid underreporting by canceling correlated errors with total energy adjustment [33]. Second, our sample was not a representative sample and could have resulted in selection bias by including a more health-aware population. Since that diet could serve as a proxy for healthy beliefs, we adjust lifestyle habits factors to mitigate this possible bias. The results did not differ from the original approach. Third, we were not able to establish a causal relationship between dietary protein and LMM given the nature of an observational study. As a result, long-term prospective studies of measuring protein density in a standard diet to prevent LMM and sarcopenia are warranted.

## 5. Conclusions

Low dietary protein intake and low vegetable protein intake were associated with a higher risk for LMM in the elderly. Since dietary protein intake is a modifiable factor, the findings of this study offer insight as to how differential protein intake influences LMM formation in our aging society.

## Figures and Tables

**Table 1:** General characteristics of the low muscle mass (LMM) and normal groups (*N* = 327).

	LMM (*n* = 94)	Normal (*n* = 233)	*p* Value
Age (years)	71.3 ± 5.0	71.6 ± 5.2	0.442
Female (%)	69.1	67.8	0.814
BMI (kg/m^2^)	23.5 ± 2.7	23.5 ± 3.2	0.841
SMI (kg/m^2^)	6.5 ± 1.2	8.5 ± 1.4	<0.001
**Lifestyle factors**			
Education (%)	88.3	93.6	0.111
Marriage (%)	70.2	73	0.616
Exercise (%)	79.8	90.1	0.019 *
Smoking (%)	3.2	7.3	0.367
Alcohol (%)	6.4	15	0.102
Coffee (%)	31.2	41.1	0.099
Tea (%)	48.9	44.6	0.773
**Major diseases**			
DM (%)	16	8.2	0.036 *
Hypertension (%)	47.9	47.6	0.97
CAD (%)	12.8	12.9	0.979
Stroke (%)	4.3	3.4	0.721
**Blood biomarkers**			
Albumin (g/L)	44.3 ± 2.2	44.5 ± 2.0	0.509
Hemoglobin (g/L)	130.0 ± 11.0	133.0 ± 13.0	0.122
Lymphocyte (%)	32.8 ± 6.4	32.0 ± 5.2	0.163
T-CHO (mmol/L)	5.5 ± 0.9	5.4 ± 1.0	0.338
AC sugar (mmol/L)	0.6 ± 0.2	0.6 ± 0.1	0.782

Abbreviations: BMI: body mass index; SMI: Skeletal muscle index; DM: diabetes mellitus; CAD: coronary artery disease; T-CHO: total cholesterol; AC sugar: fasting glucose level. * *p* < 0.05; *p* values were obtained through independent t tests or Wilcoxon rank sum tests for continuous variable and chi-square tests for categorical variables.

**Table 2:** Dietary intake of total energy, carbohydrates, proteins, fats, and vitamin supplements between the low muscle mass (LMM) and normal groups.

	LMM (*n* = 94)	Normal (*n* = 233)	*p*-Value
Total energy (Kcal/day)	1402.0 ± 358.1	1334.0 ± 315.1	0.105
Carbohydrates (g/day)	205.5 ± 58.3	200.5 ± 56.4	0.663
Carbohydrate density (%)	59.3 ± 8.7	60.2 ± 9.0	0.426
Total proteins (g/day)	50.8 ± 17.1	51.6 ± 15.0	0.528
Total protein density (%)	14.5 ± 2.9	15.5 ± 3.1	0.007 **
Vegetable protein density (%)	7.0 ± 2.4	8.1 ± 3.0	0.004 **
Animal protein density (%)	7.5 ± 2.6	7.4 ± 2.7	0.789
Fat (g/day)	40.8 ± 16.2	36.1 ± 14.2	0.012 *
Fat density (%)	26.2 ± 7.4	24.3 ± 7.7	0.028 *
Vitamin-mineral supplement (%)	36.2	46.4	0.113

* *p* < 0.05, ** *p* < 0.01.

**Table 3:** Odds ratios (and 95%CI) for total protein and vegetable protein density for low muscle mass (LMM) compared to the highest quartile ^a^.

		Total Protein Density		Vegetable Protein Density	
	Quartile	Odds Ratio (95% CIs)	*p*-Value	Odds Ratio (95% CIs)	*p*-Value
Model 1 ^b^	Quartile 1	3.11 (1.42–6.84)	0.005 **	2.50 (1.22–5.10)	0.012 *
	Quartile 2	1.99 (0.99–4.03)	0.055	2.09 (1.01–4.33)	0.047 *
	Quartile 3	1.40 (0.68–2.90)	0.362	0.99 (0.47–2.11)	0.980
Model 2 ^c^	Quartile 1	3.09 (1.40–6.82)	0.005 **	2.40 (1.17–4.94)	0.017 *
	Quartile 2	2.00 (0.98–4.05)	0.056	2.07 (0.99–4.30)	0.051
	Quartile 3	1.42 (0.68–2.95)	0.346	0.96 (0.45–2.06)	0.920
Model 3 ^d^	Quartile 1	3.03 (1.37–6.72)	0.006 **	2.34 (1.14–4.83)	0.021 *
	Quartile 2	1.86 (0.91–3.81)	0.089	2.08 (0.99–4.36)	0.051
	Quartile 3	1.33 (0.64–2.79)	0.444	0.97 (0.45–2.09)	0.944

* *p* < 0.05, ** *p* < 0.01. a. Quartiles of total protein density: Quartile 1: <13.2%, Quartile 2: 13.2%–15.0%, Quartile 3: 15.0%–17.2%, and Quartile 4: ≥17.2%. Quartiles of vegetable protein density: Quartile 1: <5.8%, Quartile 2: 5.8%–7.5%, Quartile 3: 7.5%–9.4%, and Quartile 4: ≥9.4%; b. Model 1. Adjusted for age, gender, BMI, waist, albumin, hemoglobin, lymphocyte, fat density, and total energy; c. Model 2. Adjusted for model 1 plus smoking, alcohol use, and exercise; d. Model 3. Adjusted for model 2 plus diabetes mellitus and hypertension.

## Abbreviations

The following abbreviations are used in this manuscript:

LMM	low muscle mass
SMI	skeletal muscle mass index
FFQ	Food Frequency Questionnaire
LS	least square
